# Supplementary figures and images for: Specific and versatile monoclonal antibodies for hantavirus research
Source: mSphere. 2025 Nov 25;10(12):e00612-25. doi: 10.1128/msphere.00612-25 (PMC12724165; doi:10.1128/msphere.00612-25)

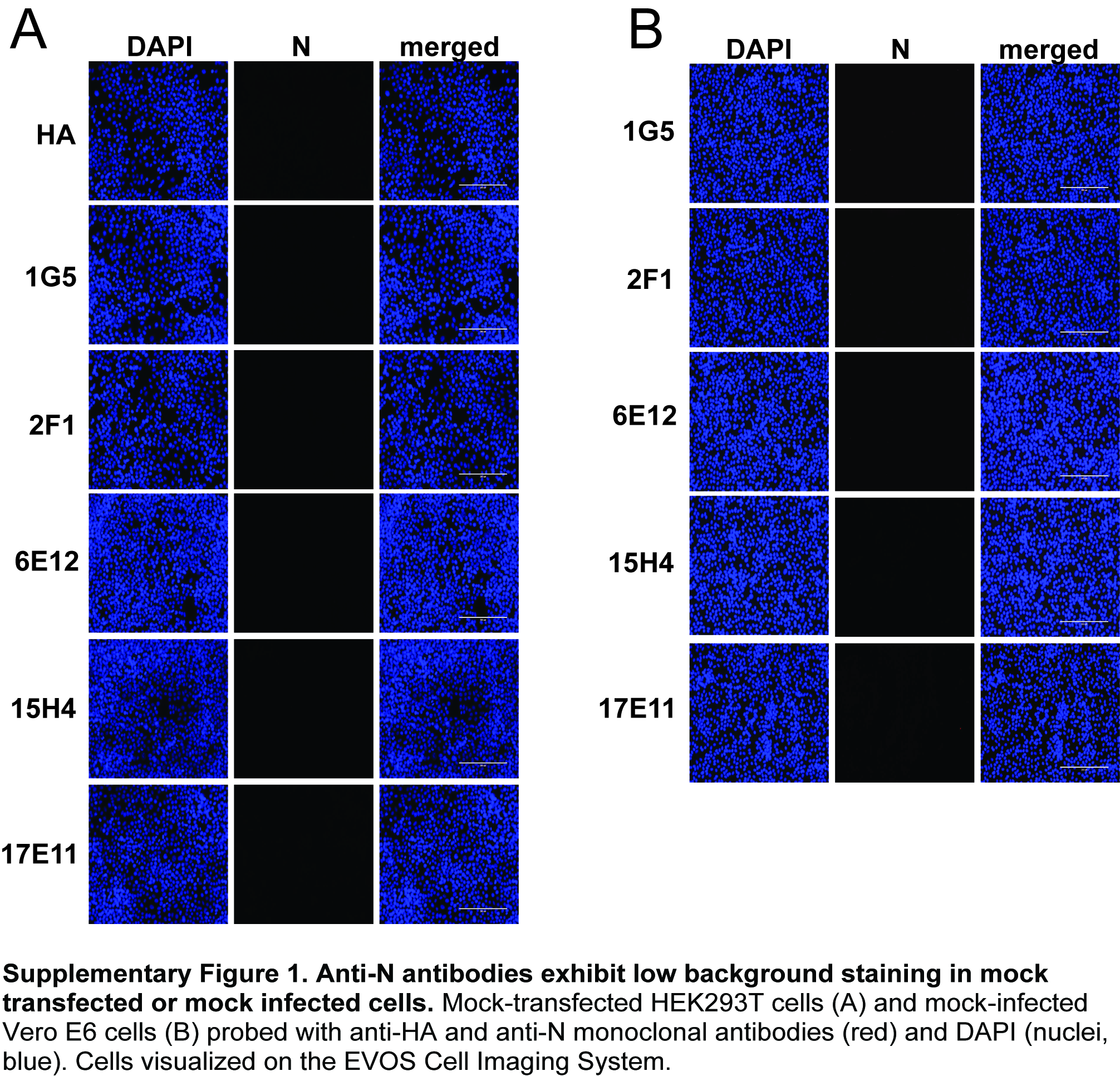

Supplement: Fig. S1 — Anti-N antibodies exhibit low background staining in mock-transfected or mock-infected cells. [file msphere.00612-25-s0001.tif]

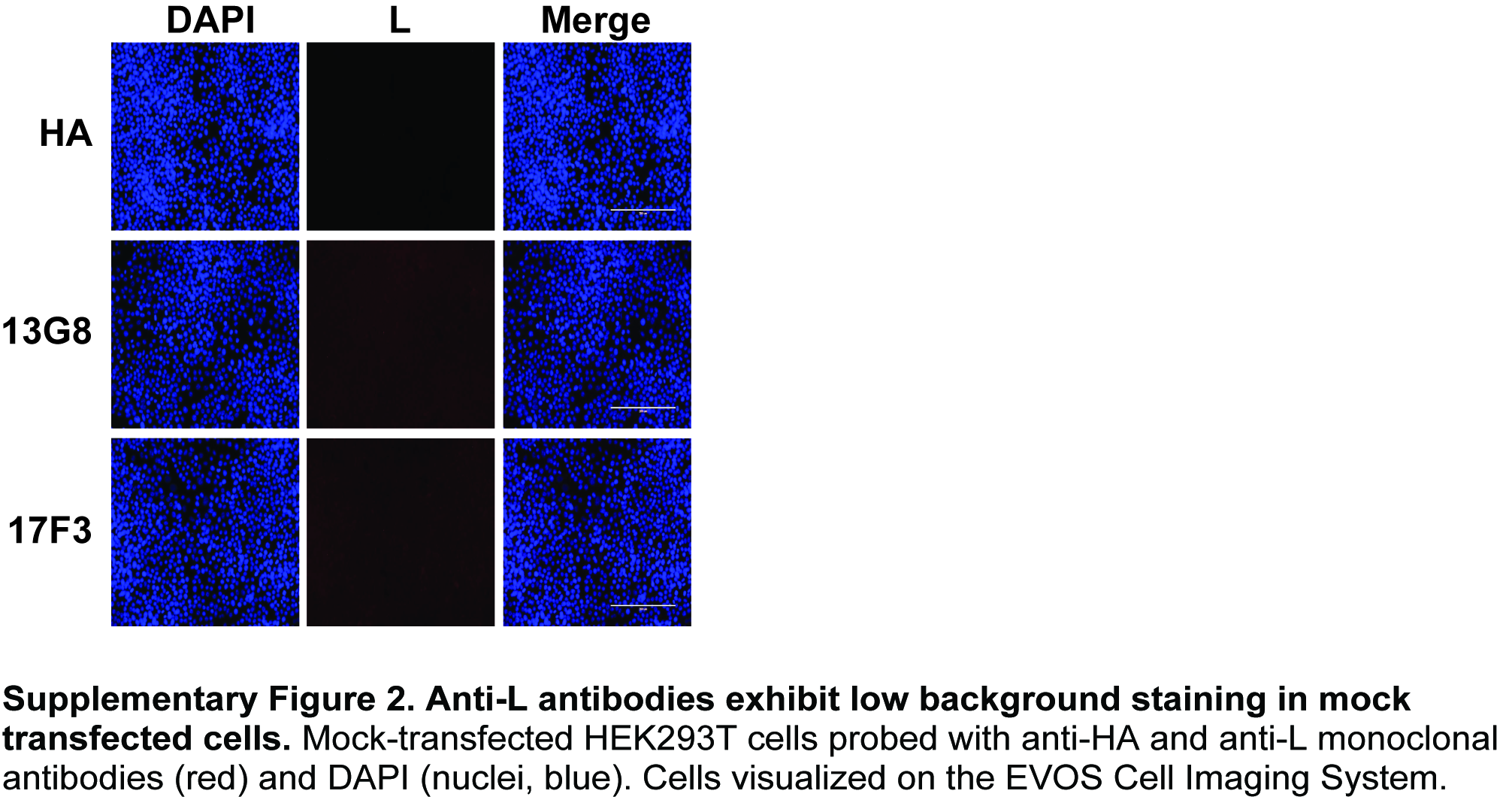

Supplement: Fig. S2 — Anti-L antibodies exhibit low background staining in mock-transfected cells. [file msphere.00612-25-s0002.tif]
